# New advances in clinical application of neostigmine: no longer focusing solely on increasing skeletal muscle strength

**DOI:** 10.3389/fphar.2023.1227496

**Published:** 2023-08-04

**Authors:** Shangkun Si, Xiaohu Zhao, Fan Su, Hongxiu Lu, Dongbin Zhang, Li Sun, Fulei Wang, Li Xu

**Affiliations:** ^1^ Shandong University of Traditional Chinese Medicine, Jinan, China; ^2^ Department of Anesthesiology, Affiliated Hospital of Shandong University of Traditional Chinese Medicine, Jinan, China

**Keywords:** α7nAChR, cholinesterase inhibitors, cholinergic anti-inflammatory pathway, inflammation, neostigmine, perioperative cognitive function

## Abstract

Neostigmine is a clinical cholinesterase inhibitor, that is, commonly used to enhance the function of the cholinergic neuromuscular junction. Recent studies have shown that neostigmine regulates the immune-inflammatory response through the cholinergic anti-inflammatory pathway, affecting perioperative neurocognitive function. This article reviews the relevant research evidence over the past 20 years, intending to provide new perspectives and strategies for the clinical application of neostigmine.

## 1 Introduction

Neostigmine has become a classic anticholinesterase drug since it was introduced in the 1930s. It inhibits acetylcholinesterase (AChE) activity, produces cholinergic effects, and enhances the transmission function of the neuromuscular junction, helping increase skeletal muscle strength. It is commonly used clinically to antagonize the residual muscle relaxation effect of non-depolarizing muscle relaxants following anesthesia surgery, and is also used to treat myasthenia gravis, postoperative functional flatulence, and urinary retention, etc., (Pohanka, 2012). In recent years, applications to modulate immune-inflammatory response through the cholinergic anti-inflammatory pathway (CAP) and affect neurocognitive function have been reported in turn, marking new progress in the clinical applications of this drug. This paper conducts a review in order to provide new evidence for the clinical application of neostigmine (The methodology of this mini-review is shown in Supplementary Material).

## 2 Pharmacological overview: cholinergic effect

Neostigmine is a carbamate derivative, belonging to the quaternary ammonium group, and is clinically used as a parasympathetic agent and cholinesterase inhibitor (ChE-Is). Its chemical structure (Kim et al., 2013) is shown in Supplementary Material. The positively charged nitrogen in the neostigmine molecule electrostatically binds to the peripheral anionic site of AChE, whereas the carbamate group in the molecule covalently binds to the serine residue in the catalytic site of the enzyme. Through serine carbamylation, the activity of AChE is reversibly inhibited. As a result, the half-life of ACh in cholinergic synapses is prolonged and the activation of nicotinic/muscarinic cholinergic receptors (nAChRs/mAChRs) is increased, ultimately producing cholinergic effects (Eldufani and Blaise, 2019). In addition, neostigmine-induced inhibition of voltage-gated potassium channels can prolong action potentials in motor neurons and thereby increase ACh release at the neuromuscular junction to increase muscle fiber contraction. Neostigmine can also directly activate postsynaptic nAChRs at the motor endplate (Liu et al., 2022a). The commonly used routes of administration are intravenous and intramuscular injection; neostigmine is difficult to be absorbed orally by the gastrointestinal tract. Due to its structural properties (quaternary amine), the drug has difficulty crossing the blood-brain barrier (BBB) and entering the central nervous system (CNS) (Luo et al., 2018).

## 3 Conventional application: classical theater

### 3.1 Muscle strength: increasing

Increasing skeletal muscle strength is the classic application of neostigmine. Neostigmine reduces the activity of AChE, thereby prolonging the half-life of ACh at the neuromuscular junction. As a result, the increased ACh-induced activation of muscle AChRs increases muscle strength. These effects make it an important alternative drug for post-anesthesia residual muscle relaxation (non-depolarizing muscle relaxation) (Fuchs-Buder et al., 2023), myasthenia gravis (Feibel, 2021), acute colonic pseudo-obstruction (Adiamah et al., 2017), constipation (Kapoor, 2008), urinary retention (Cao et al., 2022), neurotoxic snake bites (Anil et al., 2010) and other conditions or diseases that cause muscular weakness due to dysfunction of the cholinergic muscle junction.

However, since the cholinergic effect of neostigmine is limited by the amount of synaptic ACh release, it has a ceiling effect on the enhancement of muscle strength (Blobner et al., 2020). If neostigmine is administered when the neuromuscular function is normal or has fully recovered, abnormal muscle weakness may be observed due to desensitization of nAChRs caused by excessive ACh accumulation at synapses (Naguib and Kopman, 2018). Therefore, neuromuscular function monitoring is important in order to confirm the proper timing of administration (Kopman and Naguib, 2015; Phillips and Stewart, 2018). Neostigmine activates both nAChRs and mAChRs at the same time, thus it must be combined with anticholinergic drugs (such as atropine, etc.) to avoid its muscarinic side effects (arrhythmia, increased secretion, nausea, or vomiting, etc.) under different application purposes (Tajaate et al., 2018).

### 3.2 Analgesia: adjuvants

Ach is one of multiple neurotransmitters involved in regulating the production and transmission of nociceptive signals in the spinal cord. Physiological (trauma, pain, etc.) or pharmacological stimulation (activation of α2-adrenergic receptors in the spinal cord or opioid receptors in the brain stem) contribute to release of ACh. Cholinergic receptors are present in the superficial and deep dorsal horn of the spinal cord, and are involved in transmission and modulation of nociceptive signals (Eldufani and Blaise, 2019). Clinical application of neostigmine has been widely reported for perioperative adjunctive analgesia (Swain et al., 2017; Prabhakar et al., 2019). It helps to enhance the analgesic effect, prolong the analgesic time, and reduce the consumption of analgesics such as morphine, ketamine and clonidine. The drug is typically injected intrathecally or epidurally (Habib and Gan, 2006), with less use of peripheral blockade. It inhibits the activity of AChE, increasing the concentration of endogenous ACh in spinal cord synapses. Then, through mAChRs, the transmission of nociceptive signals in the spinal dorsal horn are inhibited (Duttaroy et al., 2002; Lauretti, 2015), and nociceptive signaling of various afferent fibers (such as Aβ, C and Aδ fibers) (Buerkle et al., 1998) are modulated, thus inhibiting central sensitization, and increasing the pain threshold (Naguib and Yaksh, 1994). In addition, the release of NO in the spinal cord is promoted, producing analgesic effects (Prabhakar et al., 2019).

However, neostigmine has dose-related side effects when used for adjuvant analgesia, such as nausea and vomiting, which to some extent limit its application (Prabhakar et al., 2019). Further research is needed to optimize the medication regimen.

## 4 New applications: new arena for label-off usage

### 4.1 Immune and inflammatory regulation

For a long time, immune and inflammation regulation have not been considered to be the main effects of acetylcholinesterase inhibitors such as neostigmine. However, recent evidence has shed new light on this issue.

**FIGURE 1:**
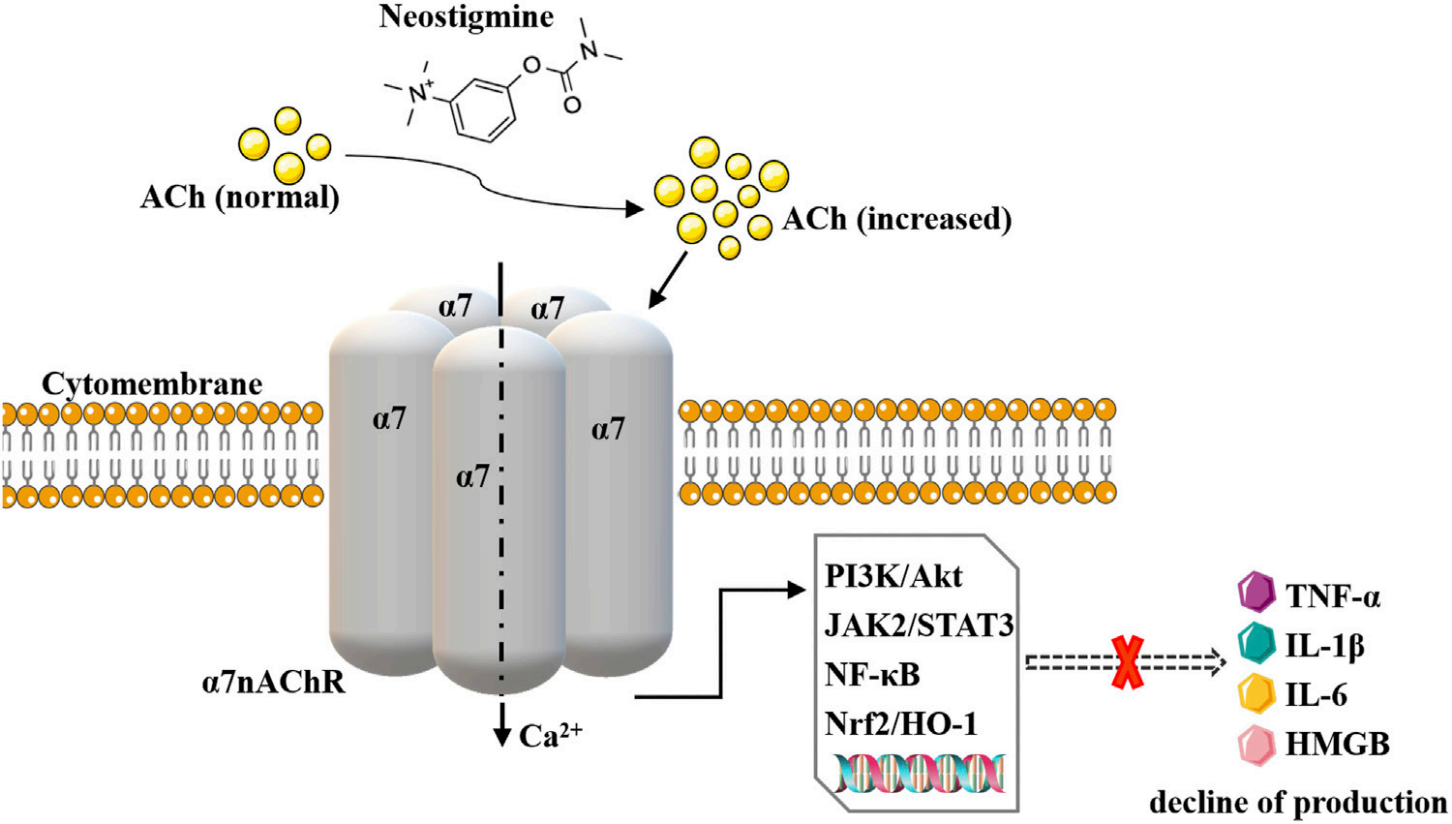
Schematic representation of neostigmine acting on CAP. Neostigmine increases the effect of ACh on α7nAChRs and amplifies CAP activity, reducing the levels of pro-inflammatory cytokines such as TNF-α, IL-1β, IL-6, and HMGB1 through PI3K/Akt, NF-kappaB, JAK2/STAT3 and other pathways.

The cholinergic anti-inflammatory pathway (CAP) is an important endogenous immunomodulatory mechanism in the body (Borovikova et al., 2000; Hoover, 2017). The local immune-inflammatory signal transmitted to the CNS nucleus tractus solitarius via the vagal afferent nerve is the driving factor of CAP. ACh released from vagal efferent nerve fibers in the inflammatory reflex pathway activates α7nAChRs expressed on immune cells (such as macrophages). Subsequently, the synthesis and release of pro-inflammatory cytokines/mediators such as tumor necrosis factor-α (TNF-α), interleukin-1β (IL-1β), interleukin-6 (IL-6), and high mobility group protein box 1 (HMGB1) are inhibited through PI3K/Akt, JAK2/STAT3, NF-κB, Nrf2/HO-1 and other pathways. Finally, the tissue damage caused by inflammation and oxidative stress is improved (Fodale and Santamaria, 2008). CAP is mainly activated by central cholinergic transmission, electrical stimulation of the vagus nerve, or cholinergic agonists. AChE-Is inhibits AChE to increase the level and duration of ACh, activate α7nAChR and amplify the activity of the CAP system to exert anti-inflammatory effects, thus improving inflammatory response damage (Nizri et al., 2006). Therefore, in terms of pharmacological mechanism, it has the potential to become an immune-inflammatory regulatory drug (Pohanka, 2014). See Figure 1.

A series of studies over the last 20 years has reported the immune-inflammatory regulatory effects of neostigmine (Miceli and Jacobson, 2003; Akinci et al., 2005; Pollak et al., 2005; Freeling et al., 2008; Hofer et al., 2008; Liu et al., 2012; Sun et al., 2012; Kalb et al., 2013; Li et al., 2014; Schneider et al., 2014; Steinebrunner et al., 2014; Zhou et al., 2014; Qian et al., 2015; Kanashiro et al., 2016; Xu et al., 2016; Abdel-Salam et al., 2018; Herman et al., 2018; Bitzinger et al., 2019; Antunes et al., 2020a; Antunes et al., 2020b; Lyu et al., 2021; El-Tamalawy et al., 2022). Its peripheral administration increases the effect of ACh on α7nAChRs and amplifies CAP activity. Through PI3K/Akt (Antunes et al., 2020a), NF-kappaB (Hofer et al., 2008; Antunes et al., 2020a), JAK2/STAT3 (Xu et al., 2016) and other pathways, it reduces the levels of pro-inflammatory cytokines such as TNF-α, IL-1β, IL-6, and HMGB1 and upregulates activity of anti-inflammatory factors such as IL-10 (Paparini et al., 2015). It can regulate the migration, recruitment, and infiltration of immune cells, inhibiting inflammatory response, oxidative stress injury and apoptosis (Liu et al., 2012). As detailed in Table 1, management with neostigmine (either prophylactic or delayed administration) triggers immunomodulatory effects in a variety of animal models (including arthritis, pancreatitis, colitis, sepsis and organ injury or failure, etc.). Some studies (Liu et al., 2012; Sun et al., 2012; Li et al., 2014; Zhou et al., 2014; Qian et al., 2015; Xu et al., 2016; Lyu et al., 2021) have found that the combination of anisodamine and neostigmine produces good anti-inflammatory effects (better than that of neostigmine alone). The mechanisms are as follows: anisodamine blocks mAChRs to prevent its non-specific parasympathetic side effects (Li et al., 2014), and indirectly enables more endogenous ACh to bind to α7nAChRs (Poupko et al., 2007; Liu et al., 2009; Zhao et al., 2011), ultimately enhancing the effect of CAP (Liu et al., 2009; Xu et al., 2011; Zhao et al., 2011; Zhang et al., 2023).

**TABLE 1:** Neostigmine regulation of immune and inflammatory responses.

Author/Year	Model	Mode/dose	Mechanism/pathway	Inflammation-related index
Miceli and Jacobson (2003)	Acute dinitrobenzene sulfonic acid colitis Rats	s.c. 50 μg/kg	-	MPO (proximal to the anus) and combined macroscopic colonic damage score↓
Akinci et al. (2005)	Septic shock Mice	i.p. 0.1 or 0.3 mg/kg every 6 h for 3 days	-	Interstitial inflammation in the lungs↓; Vacuolar degeneration in the liver↓; Total liver injury↓
Pollak et al. (2005)	LPS-induced inflammation Mice	i.p. 0.15 mg/kg	AChE	IL-1β (hippocampus and blood)↓
Hofer et al. (2008)	Sepsis induced by cecal ligation and puncture Mice	i.p. 80 μg/kg three times daily for 3 days	NF-κB	Pulmonary neutrophil invasion↓; MPO activity (lung)↓; Survival↑
Freeling et al. (2008)	Heart with pressure overload Rats	i.p. 3 or 6 μg/kg per day for 14 days	-	Heart tissue: TNF-α↓, IL-10↑; Cardiac hypertrophy↓; Ventricular function↑
Sun et al. (2012)	Endotoxic shock Mice/Hemorrhagic shock Dogs	Mice: i.p. 12.5 or 25 or 50 μg/kg at 0, 3, and 6 h after LPS exposure; Dogs: i.v. 5.25 μg/kg	α7nAChR	Serum: TNF-α and IL-1β↓; Survival rate↑; Liver: damage, infiltration by inflammatory cells and putrescence of hepatic cells↓
Liu et al. (2012)	Ischemic stroke Rats	unknow 40 or 80 μg/kg	α7nAChR	Serum: TNF-α and IL-6↓; Neurological deficit score↓; Infarct size↓; Ischemic penumbra: cleaved caspase 8, Bad, and Bax↓, Bcl-2 and Bcl-xl↑
Kalb et al. (2013)	Laparotomy combined with LPS Rats	i.p. 100 μg/kg before the LPS-application, and s.c. 100 μg/kg for 3 times	-	IL-1β (cortex and hippocampus)↓; IL-1β and TNF-α (spleen and plasma)↓
Schneider et al. (2014)	Necrotizing pancreatitis Rats	i.p. loading dose of 0.05 mg/kg and then 0.0124 mg/kg per hour for 9 h	-	MPO (pancreatic tissue) and HMGB1 (serum)↓; Pancreatic morphological damage↓
Li et al. (2014)	Biliary drainage and partial hepatectomy Rats	i.p. 50 μg/kg twice per day for 2 days	-	Remnant livers: TNF-α, IL-1β, IL-6, MCP-1 and MIP-1α↓; Serum: TNF-α and IL-1β↓; Migration and infiltration of neutrophils and the hepatocyte injury↓
Zhou et al. (2014)	Collagen-induced arthritis Mice	i.p. 50 μg/kg per day for 10 days	-	Serum: TNF-α, IL-6 and IL-1β↓; Anti-type II collagen specific antibodies IgG and IgG_2a_↓; Arthritis index and joint swelling↓
Steinebrunner et al. (2014)	Acute liver failure induced by acetaminophen Mice	i.p. 80 μg/kg either 1 h before or 1, 7, 12, 24 h after application of APAP	-	Serum: TNF-α and IL-1β↓; Hepatocellular damage↓(LDH, ALT↓); Histopathological liver damage and apoptosis↓; Survival↑
Qian et al. (2015)	Ischemic stroke Rats/Mice	i.v. 40 μg/kg	α7nAChR	Serum: TNF-α, IL-6 and IL-1α↓; Infarct size and neurological deficit score↓; Ischemic penumbra: Bad and Bax↓, Bcl-2 and Bcl-xl↑
Xu et al. (2016)	Acute lethal crush syndrome Rats/Rabbits	Rats: i.p. 40 μg/kg; Rabbits: i.p. 20 μg/kg	α7nAChR; JAK2-STAT3	24 h survival rate↑; Compressed muscle: TNF-α, IL-6 and IL-10↓; Serum and compressed muscle: H_2_O_2_, MPO and NO↓
Kanashiro et al. (2016)	Antigen-Induced Arthritis Mice	s.c. 12.5, 25 and 50 μg/kg twice a day for 7 days	-	Neutrophil recruitment in the knee joint↓
Herman et al. (2018)	Immune stress Ewes	i.v. 0.5 mg	α7nAChR	IL-1β (serum)↓; Hypothalamus: IL-1β, IL-6, and TNF-α↓
Abdel-Salam et al. (2018)	Acute malathion exposure Rats	i.p. 200 or 400 μg/kg	AChE; BChE	GSH (brain)↑; Neuronal degeneration (cortex and hippocampus)↓ GFAP(hippocampus)↓; Liver damage↓
Bitzinger et al. (2019)	CLP-induced sepsis Rats	i.p. 75 μg/kg four times over 24 h	-	ROS production and CD11b upregulation↓
Antunes et al. (2020a)	Allergic asthma Mice	i.p. 80 μg/kg per day for 3 days	AChE; α7nAChR; NF-κB; PI3K/Akt	Lung tissue: IL-4, IL-5, IL-13, IL-1β, TNF-α and ROS↓, CAT↑; EPO activity in BAL↓; Peribronchial and perivascular inflammatory infiltrates↓
Antunes et al. (2020b)	Allergic asthma Mice	i.p. 80 μg/kg per day for 3 days	AChE	Leukocyte recruitment (BAL)↓; Leukocyte infiltrate (lung)↓; ROS and CAT (cerebral cortex)↓; SOD/CAT ratio↑
Lyu et al. (2021)	Biliary obstruction Rats	i.p. 50 μg/kg per day for 7 days	-	Serum: CRP, TNF-α and IL-1β↓; Liver function↑(ALT, AST, TB, DB, and GGT↓)
El-Tamalawy et al. (2022)	Sepsis or septic shock Patients	i.v. 0.2 mg/h for 120 h	-	SOFA and Progression from sepsis to septic shock↓; Incidence of shock reversal↑

Tip: MPO, myeloperoxidase; Bad and Bax are pro-apoptosis protein; MCP-1, monocyte chemotactic protein 1, a protein secreted by astrocytes that promotes inflammation; MIP-1α is a macrophage inflammatory protein; Bcl-2, and Bcl-xl are anti-apoptosis protein; GSH, glutathione; GFAP, glial fibrillary acidic protein; BAL, bronchoalveolar lavage; CAT, catalase; EPO, eosinophil peroxidase; SOFA, sequential organ failure assessment.

Contrary to the findings above, however, some studies report that neostigmine does not modulate immune and inflammatory responses (Tyagi et al., 2010; Kox et al., 2011; Leib et al., 2011; Zhang et al., 2016). However, the reasons for the ineffectiveness have not been fully elucidated. Meanwhile, studies have reported that while neostigmine plays an immune-inflammatory regulatory role through CAP (activation of α7nAChRs), parasympathetic side effects may be observed (due to activation of mAChRs) (Akinci et al., 2005), requiring combination with anticholinergic drugs (e.g., anisodamine, the collaborator mentioned above) or dosage control, thus its application is somewhat limited. In addition, the above anti-inflammatory data on neostigmine are mostly nonclinical studies, and only one study reported its clinical anti-inflammatory effect in septic shock patients. Clinical evidence that neostigmine affects other inflammatory diseases is scarce, which limits the external validity and clinical anti-inflammatory application promotion. More large-sample and multi-center clinical studies are required to provide evidence for the regulatory effect of neostigmine on inflammation.

### 4.2 Perioperative neurocognitive protection

Perioperative neurocognitive disorders (PND) refer to the decline and deterioration of abilities in multiple cognitive domains during the perioperative period, including preoperative cognitive dysfunction, postoperative delirium (POD), and postoperative cognitive dysfunction (POCD). (Evered et al., 2018). The pathological role of CNS inflammatory response caused by anesthesia and surgery in PND has been widely reported (Liu et al., 2022b). Anesthesia and surgery (tissue damage or pathogenic attack) induce activation of the body’s immune-inflammatory system, resulting in a rapid increase in levels of pro-inflammatory cytokines (e.g., IL-1β, TNF-α, and IL-6) in a short period of time, causing peripheral local inflammation. Anesthesia and surgery can also increase the permeability of the BBB (Glumac et al., 2019). Inflammatory factors enter the CNS from the periphery through the BBB (Banks et al., 1995), and at the same time, peripheral inflammatory signals are transmitted to the brain through the afferent nerve. Then immune-related cells (such as astrocytes and microglia) in the hippocampus and other regions of the brain are activated, and pro-inflammatory cytokines such as IL-1β are released, thus causing a central immune-inflammatory response (Needham et al., 2017). The whole process interferes with the activity of neurons and synaptic transmission in the cerebral cortex or hippocampal region of the brain, ultimately affecting perioperative cognitive function (Cibelli et al., 2010; Fidalgo et al., 2011; Liu and Yin, 2018; Liu et al., 2022b). Neostigmine, as the most typical cholinesterase inhibitor in used general anesthesia, is often used to antagonize postoperative residual neuromuscular blockade and is occasionally used as an adjunct to perioperative analgesia. At the same time, cholinergic system activity and CAP activity can be increased through the cholinergic effect of neostigmine. Therefore, whether neostigmine could improve CNS immune-inflammation impairment by increasing cholinergic system activity and through mechanisms such as CAP, and thus be an exposure factor for improving perioperative cognitive function, is a clinical question worth investigating.

#### 4.2.1 Neostigmine on central anti-inflammation and neuro-protection: nonclinical studies

Animal experiments have shown that neostigmine inhibits AChE, increases cholinergic system activity and neurotransmission (Pollak et al., 2005), and then activates CAP, regulates the activation level of CNS immune cells such as microglia and astrocytes, and reduces the expression of pro-inflammatory cytokines such as IL-1β (Kalb et al., 2013), thereby attenuating or delaying the inflammatory response, oxidative stress and neuronal degeneration in the cerebral cortex and hippocampus of the surgical stress model rats (Abdel-Salam et al., 2018), maintaining synaptic plasticity (Tozzi et al., 2015), and finally exerting central immune-inflammatory response regulation and neuroprotective effects (Antunes et al., 2020b). However, the data above only reported the central anti-inflammatory and neuroprotective effects of neostigmine, without quantitative evaluation of neurocognitive function changes in animals.

#### 4.2.2 Neostigmine on perioperative neurocognitive function: clinical studies

An earlier study by Prohovnik et al. (1997) found that intravenous administration of neostigmine (11 μg/kg) exhibited a reversal effect on scopolamine-induced memory deficits in healthy subjects (no difference from the effect of 22 μg/kg of physostigmine, which can be transferred into the CNS). One case report showed that intravenous neostigmine improved patients’ delirium symptoms while treating postoperative acute colonic pseudo-obstruction (Lankarani-Fard and Castle, 2006). Zhu et al. found that the incidence of early postoperative cognitive decline in elderly patients undergoing radical resection of gastrointestinal cancer following intravenous injection of 0.04 mg/kg neostigmine in PACU was significantly lower than in the control group (Zhu et al., 2020). Cozanitis et al. showed that the postoperative Wechsler Memory Scale scores of elderly cataract surgery patients in the neostigmine group were similar to those in the galantamine group (a type of AChE-Is, acting on the CNS, commonly used in neurodegenerative diseases such as Alzheimer’s disease and Parkinson’s disease) (Cozanitis et al., 2012). However, Batistaki et al. came to the opposite conclusion: no significant difference in POCD incidence in middle-aged and elderly surgical patients in the neostigmine group compared with sugammadex (Batistaki et al., 2017); and Liu et al. indicated that application of neostigmine in patients undergoing colon cancer surgery did not reduce the incidence of POD (Liu et al., 2022a). The reasons are speculated as follows: i) Neostigmine is mainly used postoperatively, with rapid half-life elimination and short duration of action (Carron et al., 2016), thus making it difficult to account for the influence of anesthetic surgical factors on the CNS in a short period of time; ii) Neostigmine is often combined with anticholinergic drugs such as atropine to avoid its non-specific parasympathetic adverse reactions, while anticholinergic effects antagonize the cholinergic and CAP effects of neostigmine (Zwart and Vijverberg, 1997; Gonzalez-Rubio et al., 2006). The clinical data above show the potential of neostigmine to improve perioperative neurocognitive function. However, there was heterogeneity in the type of surgery involved in these studies, and the quantitative assessment tools of cognitive function were not consistent. At the same time, some studies did not provide sample size estimation basis. This limited heterogeneity control may therefore introduce bias, and the results need to be viewed with caution.

Many issues need to be explored before the effects of neostigmine on central anti-inflammatory and neuroprotective effects are confirmed. It is generally accepted that drugs need to be present in the central compartment to have CNS effects (Pavlov et al., 2009; Noori et al., 2012; Herman et al., 2019). As a quaternary ammonium compound, however, neostigmine does not readily cross the BBB and stays in the peripheral compartment when delivered via non-central routes of administration. Thus, it is difficult to understand how peripheral administration of neostigmine modulates inflammation in the CNS. Through literature review and collation, the mechanisms are speculated as follows. i) Increased BBB permeability: Anesthesia and surgery, inflammatory response, stress, and other factors increase BBB permeability (Friedman et al., 1996; Beck et al., 2003; Danielski et al., 2018; Glumac et al., 2019). Peripheral neostigmine may enter the CNS through the damaged BBB to function (Zhu et al., 2020). However, the results of current studies on the effect of peripheral administration of neostigmine on central AChE activity under certain specific conditions are controversial (Pollak et al., 2005; Kalb et al., 2013; Zhang et al., 2016; Abdel-Salam et al., 2018; Dubrovskii et al., 2018; Antunes et al., 2020b). Therefore, the available evidence is still inconclusive about whether neostigmine can directly play a corresponding role through the BBB into the CNS in some special cases. ii) Improving peripheral inflammation: Peripheral inflammatory signals are transmitted to the CNS via vagal afferent nerves, while peripheral pro-inflammatory cytokines reach the brain parenchyma through the BBB (due to increased BBB permeability or active transport mechanisms). Both the nervous signals and humoral factors to some extent directly or indirectly cause CNS immune-inflammatory responses (Banks et al., 1995; Needham et al., 2017; Noll et al., 2017). Neostigmine reduces the peripheral inflammatory response level and expression of pro-inflammatory cytokines. Then, the transmission of peripheral inflammatory signals to the central system is attenuated (Herman et al., 2017; Herman et al., 2018), and the CNS is less affected by peripheral pro-inflammatory cytokines passing through the BBB (peripheral pro-inflammatory cytokines need to be enriched to a critical level in order to affect the CNS (Antunes et al., 2020b)), thus reducing the central immune-inflammatory response (Pollak et al., 2005; Steinman, 2010; Kalb et al., 2013) (The relationship between neostigmine and neuroprotective effects is shown in Supplementary Material). iii) Other potentially significant factors: neostigmine may be involved in changes in cognitive function by modulating the peripheral cholinergic cerebrovascular circulation (Kocsis et al., 2014). This view is based on the functional basis of high energy and oxygen consumption in the brain. Contrary to most people’s perception, carbamate compounds such as neostigmine may exert CNS effects in certain circumstances - an important point that should not be ignored in the evaluation of the effects of neostigmine!

## 5 Conclusion and perspective

Recent studies have shown that the classic cholinergic effect of neostigmine may play a new role in immune-inflammatory regulation and perioperative neurocognitive protection through CAP. However, the function, downstream regulatory targets and transduction pathways of CAP are still not completely clear. Current studies on neostigmine in immune-inflammation regulation and perioperative neurocognitive protection are mostly animal experiments, and more clinical studies are needed for verification. Considering the non-specific parasympathetic side effects of neostigmine under different application purposes, further research on the reasonable medication regimen is required. It is believed that with deep follow-up research, new and comprehensive neostigmine usage strategies will be observed.
