# Supplementary material for: New advances in clinical application of neostigmine: no longer focusing solely on increasing skeletal muscle strength
Source: Front Pharmacol. 2023 Aug 4;14:1227496. doi: 10.3389/fphar.2023.1227496 (PMC10436336; doi:10.3389/fphar.2023.1227496)

***Supplementary Material***

**New advances in clinical application of neostigmine: No longer focusing solely on resuscitating muscle strength**

**Shangkun Si^1^†, Xiaohu Zhao^1^†, Fan Su^2^*, Hongxiu Lu^2^*, Dongbin Zhang^2^*, Li Sun^2^, Fulei Wang^1^, Li Xu^1^**

1.Shandong University of Traditional Chinese Medicine, Jinan, China

2.Department of Anesthesiology, Affiliated Hospital of Shandong University of Traditional Chinese Medicine, Jinan, China

†These authors have contributed equally to this work.

*** Correspondence:**

Corresponding Author: [zhangdbzunyi@163.com](mailto:zhangdbzunyi@163.com)(Dongbin Zhang), 15306418330@163.com(Hongxiu Lu) and [boatsail@126.com](mailto:boatsail@126.com)(Fan Su).

**1. Supplementary Figure**


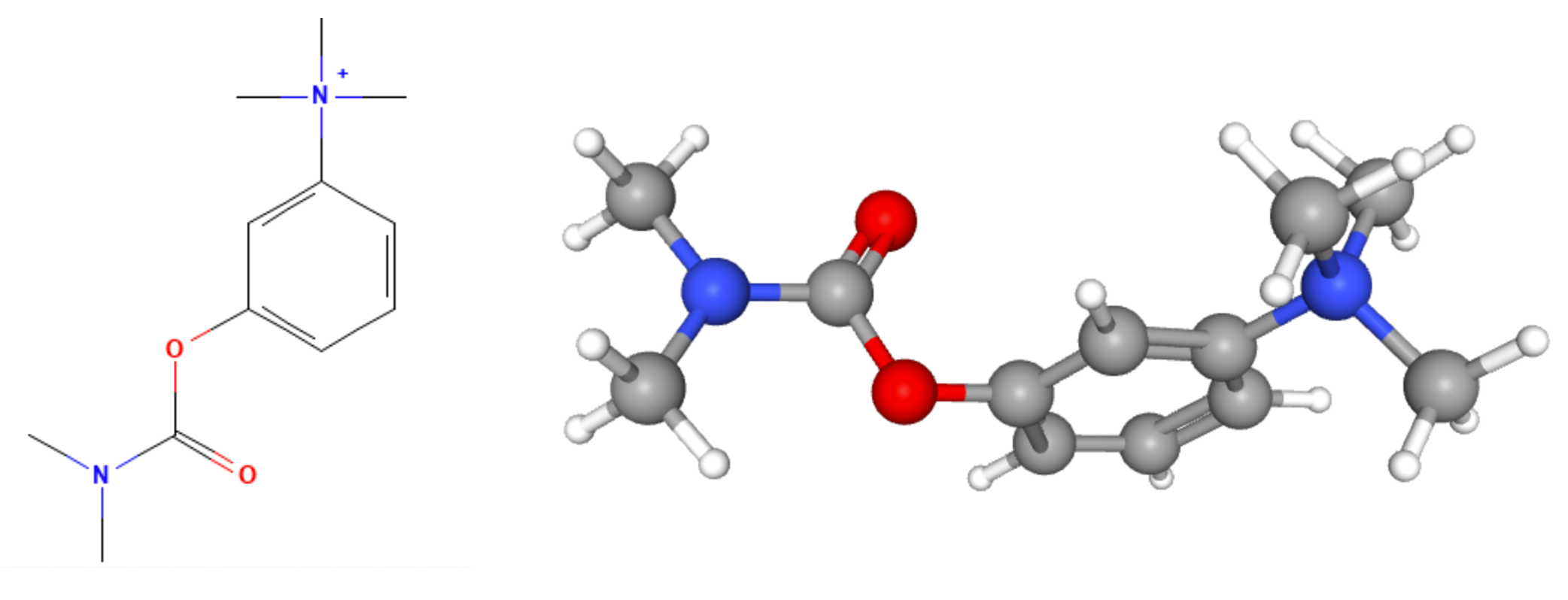


2D/3D structure of neostigmine.

**2.** **Methodology**

2.1 Objectives

This article reviews the relevant research evidence in the past 20 years intending to provide

new perspectives and strategies for the application in immune-inflammatory regulation and perioperative neurocognitive function of neostigmine.

2.2 Search strategy

Databases such as MEDLINE, Web of Science, PubMed, Embase, Cochrane Library were searched (the last 20 years). The search retrieved all articles using the MESH or free-text word terms : 'neostigmine', 'prostigmine', 'proserin', 'neostigmine bromide', 'cognitive', 'cognition', 'POD', 'POCD', 'PND', 'cholinergic anti-inflammatory pathway', 'α7nAChR', 'inflammatory', 'cytokine', 'inflammation', and 'neuroinflammation'. Variations of the terms were also included in the search, and the reference lists of relevant articles for potentially eligible studies were manually checked.

2.3 Eligibility criteria

Inclusion criteria : Studies of neostigmine in immune-inflammatory regulation and perioperative neurocognitive function, without restrictions of language or research type.

Exclusion criteria : The original text was not available or studies where outcome indicators were incomplete.

2.4 Outcome indicators

The mechanism of action of neostigmine in clinical/non-clinical immune-inflammatory regulation, and the regulatory effect of neostigmine on inflammation-related indicators. The effect of neostigmine on perioperative cognitive function changes (Cognitive assessment scales or incidence of PND).

2.5 Data extraction

Note Express v3.5.0 was used to manage the included research literature. Office Excel was used to make tables, and to summarize, de-duplicate, screen and extract research data from the research literature. Two independent people screened literatures through titles and abstracts, and then re-screened them based on the full text. The data, such as authors’ names, years of publication, sample size, interventions, and outcome indicators were extracted from the final publications of the studies. In case of disagreement, it was referred to the corresponding author for arbitration.

From: Page MJ, McKenzie JE, Bossuyt PM, Boutron I, Hoffmann TC, Mulrow CD, et al. The PRISMA 2020 statement: an updated guideline for reporting systematic reviews. BMJ 2021;372:n71. doi: 10.1136/bmj.n71

**3.** **Relationship between neostigmine and PND**

| Author/Year | Type | Outcome/Conclusion |
| --- | --- | --- |
| Banks et al., 1995 ^(64)^ | Review | Blood-borne cytokines could potentially affect brain function. |
| Glumac et al., 2019 ^(63)^ | Review | The inflammatory response plays an important role in POCD development. |
| Liu et al., 2022 ^(62)^ | Review | Neuroinflammation has been characterized as one of the major causes, especially in the elderly patients. |
| Liu et al., 2018 ^(66)^ | Review | After surgery, alarmins are leaked from the injury sites and proinflammatory cytokines are increased in the peripheral circulation. Neurons in the hippocampus, which is responsible for learning and memory, can be damaged by cytokines transmitted to the brain parenchyma. |
| Pollak et al., 2005 ^(31)^ | Nonclinical study | Neostigmine significantly attenuated the production of IL-1β in the hippocampus and blood. |
| Cibelli et al., 2010 ^(68)^ | Nonclinical study | Surgery caused hippocampal-dependent memory impairment that was associated with increased plasma cytokines, as well as reactive microgliosis and IL-1β transcription and expression in the hippocampus. |
| Fidalgo et al., 2011 ^(67)^ | Nonclinical study | Surgery caused hippocampal-dependent memory impairment, which was associated with increased levels of IL-1β both in plasma and hippocampus. |
| Kalb et al., 2013 ^(36)^ | Nonclinical study | Surgery accompanied by LPS-treatment led to increased IL-1β, IL-1 and TNF-α in the cortex and hippocampus but was significantly reduced by neostigmine. |
| Abdel-Salam et al., 2018 ^(45)^ | Nonclinical study | Treatment with neostigmine + atropine afforded protection against the deleterious effects of acute malathion on the brain(neuronal degeneration). |
| Antunes et al., 2020 ^(48)^ | Nonclinical study | Neostigmine may play a protective role in nerve damage caused by asthma. |
| Prohovnik et al., 1997 ^(70)^ | Clinical study | Neostigmine exhibited a reversal effect on scopolamine-induced memory deficits in healthy subjects. |
| Lankarani-Fard et al., 2006 ^(71)^ | Clinical study | Neostigmine improved patients' delirium symptoms while treating postoperative acute colonic pseudo-obstruction. |
| Cozanitis et al., 2012 ^(73)^ | Clinical study | The postoperative Wechsler Memory Scale scores of elderly cataract surgery patients in the neostigmine group were similar to those in the galantamine. |
| Batistaki et al., 2017 ^(74)^ | Clinical study | Compared with sugammadex, there was no significant difference in the incidence of POCD in middle-aged and elderly surgical patients in the neostigmine group. |
| Zhu et al., 2020 ^(72)^ | Clinical study | Incidence of early postoperative cognitive decline in elderly patients undergoing radical resection of gastrointestinal cancer after neostigmine was significantly lower than that in the control group. |
| Liu et al., 2022 ^(4)^ | Clinical study | Neostigmine in patients undergoing colon cancer surgery did not reduce the incidence of POD. |

Tip: CNS inflammation plays an important role in the pathogenesis of PND, and neostigmine can prevent and manage PND by improving central inflammation. Neostigmine reduces the level of peripheral inflammatory response and the expression of pro-inflammatory cytokines. Then the transmission of peripheral inflammatory signals to the central system will be attenuated, and CNS will be less affected by peripheral pro-inflammatory cytokines passing through the BBB, reducing the central immune-inflammatory responses.


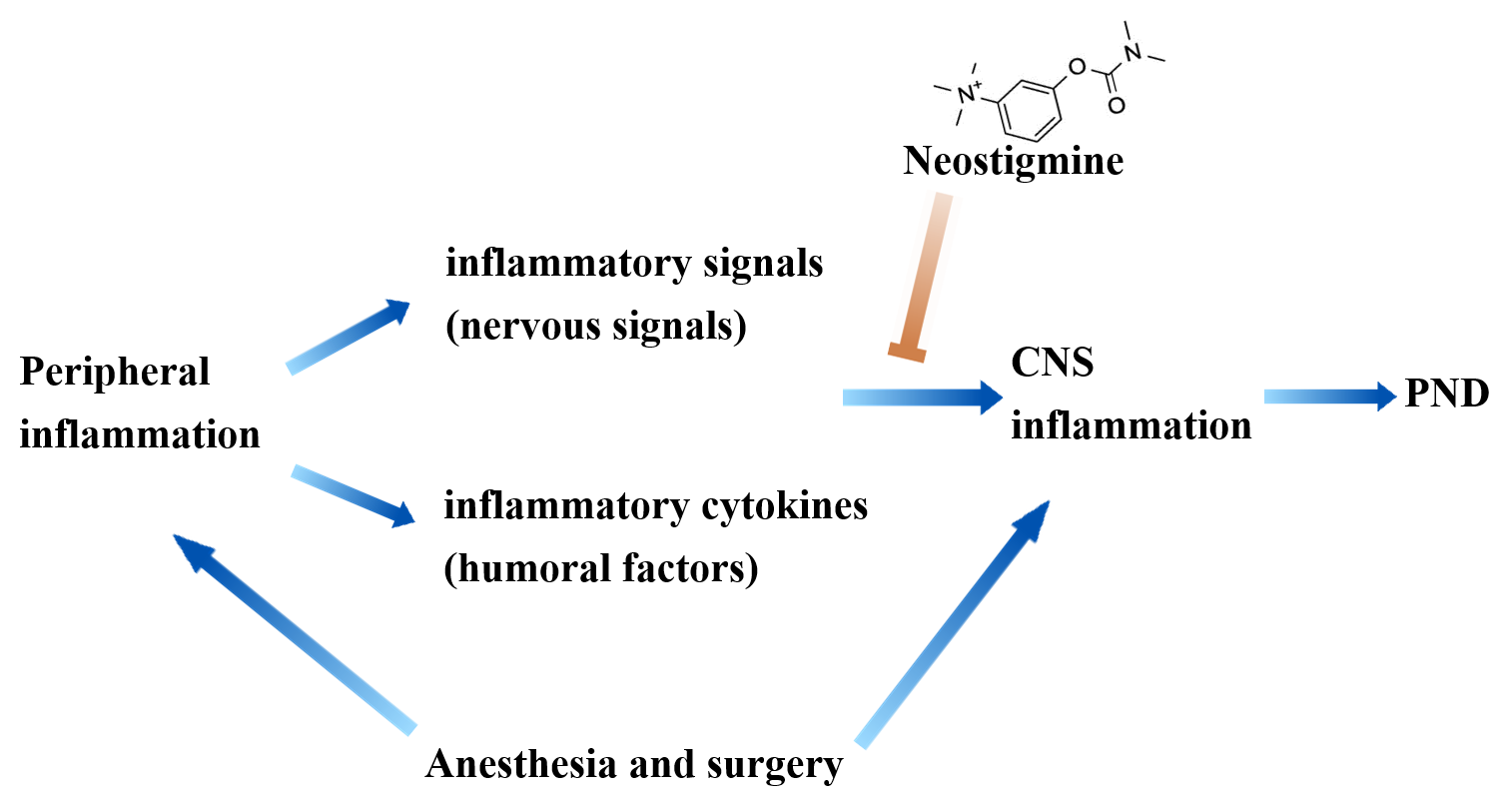

Supplement: Supplementary file 1 [file Table1.DOCX]
